# Evaluating the Therapeutic Efficacy of Si-Wu-Tang Decoction and Concentrated Extract in Follicular Maldevelopment-Related Menstrual Disorders Through Pharmacokinetic/Pharmacodynamic Studies

**DOI:** 10.3389/fphar.2020.01245

**Published:** 2020-09-04

**Authors:** Chia-Jung Lee, Alinafe Magret Kapelemera, Yi-Zhe Tsai, Ching-Tzu Lee, Ming-Yi Xu, Ching-Chiung Wang

**Affiliations:** ^1^ Graduate Institute of Pharmacognosy, College of Pharmacy, Taipei Medical University, Taipei, Taiwan; ^2^ PhD Program in Clinical Drug Development of Herbal Medicine, College of Pharmacy, Taipei Medical University, Taipei, Taiwan; ^3^ Traditional Herbal Medicine Research Center, Taipei Medical University Hospital, Taipei, Taiwan; ^4^ Department of Traditional Chinese Medicine, Taipei Municipal Wanfang Hospital, Taipei, Taiwan; ^5^ School of Medicine, College of Medicine, Taipei Medical University, Taipei, Taiwan; ^6^ School of Pharmacy, College of Pharmacy, Taipei Medical University, Taipei, Taiwan

**Keywords:** Si-Wu-Tang, ferulic acid, menstrual disorders, follicular maldevelopment, pharmacokinetic/pharmacodynamic

## Abstract

Si-Wu-Tang (SWT), a traditional Chinese formula, is commonly used to relieve menstrual discomfort and climacteric syndrome. Water decoction (WD) and concentrated herbal extract (CHE) are the two most common formulations of traditional Chinese medicine (TCM). However, few studies have reported the equivalency of these two formulations. In this study, 23 healthy volunteers were included to determine the pharmacokinetic (PK) equivalent dosage of WD and CHE, and 25 infertile women with follicular maldevelopment to evaluate the pharmacodynamic (PD) effects on menstrual disorders. The randomized, two-way crossover comparative PK study of SWT-WD and SWT-CHE analyzed the active component, ferulic acid. The results showed that clinical doses of 170 mL SWT-WD and 18 g SWT-CHE produced the same amount of ferulic acid in the blood. The PD study showed that patients who took both of these formulations had an initial luteinizing hormone/follicle-stimulating hormone ratio of <1; however, the value returned to normal and their symptoms all improved after taken SWT. Our results showed that WD and CHE, both prepared from 40 g of SWT, displayed bioequivalence upon PK/PD analysis.

## Introduction

Traditional Chinese medicine (TCM) has been developed and used for over 2,500 years. TCM is still widely used in Taiwan and is beginning to receive global recognition. A decoction is an important dosage form of TCM. Boiling has a great influence on its clinical efficacy. Decoctions are difficult to prepare and transport, and it is challenging to maintain quality control. Moreover, a commonly commercial extraction method might only obtain 40–70% of the active ingredients (Schulzki et al., 2017). This results in loss of medicinal resources and may reduce clinical efficacy. Following the advancement of pharmaceutical technology, concentrated herbal extracts (CHEs) have been used since 1963. Depending on the characteristics of the medicinal materials, a pharmaceutical factory may use different procedures to accurately control the amount of water, temperature, and time taken to produce products with consistent quality. According to the announcement No. 84056272 (August 31, 1995) and No. 89037929 (June 29, 2000) from Ministry of Health and Welfare, Department of Chinese Medicine and Pharmacy and Taiwan Herbal Pharmacopeia, recommended doses of crude drugs for decoction range from 20 to 40 g/day (Ministry of Health and Welfare, 2018). In addition, Japan also has extensive and frequent experiences in using concentrated formulations. Japanese Kampo medicine has also developed varieties formulations, such as granules, tablets and powders. Among them, granules are the most commonly used in Taiwan and Japan. The amount differs because the CHE still needs to be combined with other drugs. However, there are many questions with regards to the bioavailability and efficacy of decoctions and CHEs that remain unanswered (Lai et al., 2013).

The proportion of infertile women has increased with increase in childbearing age. Infertility is associated with many factors including nutrition, disease conditions, and malformation of the uterus (Ried, 2015; Hanson et al., 2017). Infertility affects women worldwide. Ovulation is the release of eggs from the ovaries, and the ovarian follicles rupture to release secondary oocytes. However, ovarian follicular maldevelopment means that follicular growth does not reach maturity (Tal et al., 1985). Polycystic ovarian syndrome (PCOS), one of the most common endocrine disorders in women, is characterized by oligo-anovulation, clinical or biochemical hyperandrogenemia, and polycystic ovaries on ultrasonography. An alteration in gonadotropin-releasing hormone secretion causes an increase in luteinizing hormone (LH) secretion but normal follicle-stimulating hormone (FSH) secretion remains unaffected. This pattern of secretion results in an abnormal LH/FSH ratio in many patients. Hence, LH and FSH are widely accepted as specific endocrine profile markers (Le et al., 2019; Park et al., 2019; Sidra et al., 2019).

Pallor, irregular menstrual cycles, and a lack of energy are all considered general indicators of a blood deficiency in TCM (Chang et al., 2016). Blood deficiency is common in women and can be associated with backaches, premenstrual spotting, abdominal pain, and can be a cause of female infertility or ovarian follicular maldevelopment. Si-Wu-Tang (SWT), a well-regarded ancient TCM, was first recorded in *Tai Ping Huei Min Ho Chi Chu Fang* of the Sung dynasty (AD 1,107–1,110). SWT can address blood deficiencies and has been shown to promote a normal menstrual cycle in some cases of chronic blood deficiency with menstrual irregularities (Huang et al., 2019; Lin et al., 2019). According to TCM theory, SWT is a foundational blood tonic formula composed of four Chinese herbs: Angelica Radix (the root of *Angelica sinensis* (Oliv.) Diels, *Dang Gui*), Chuanxiong Rhizoma (rhizome of *Ligusticum chuanxiong* Hort., *Chuan Xiong*), Paeoniae Radix (the root of *Paeonia lactiflora* Pall., *Shao Yao*), and Rehmanniae Radix (the processed root of *Rehmannia glutinosa* Libosch., *Shu Di Huang*). The herbs *Shu Di Huang* and *Shao Yao* tonify blood; *Dang Gui* and *Chuan Xiong* regulate the Qi of the blood to dispel and prevent blood stasis, which often develops from chronic blood deficiency (Dai et al., 2002; Fang et al., 2013). In the blood-deficiency model caused by bloodletting, mice that were administered SWT exhibited an increase in white and red blood cells as well as total number of bone marrow nucleated cells (Sun et al., 2016).

Ferulic acid, ligustilide, paeoniflorin, senkyunolide A, and catapol are usually used as quality control standers in Si-wu-Tang. However, ligustilide and senkyunolide A are hard dissolved in the water. And there was few research to evaluate the follicular maldevelopment of paeoniflorin. Furthermore, our previous study determined that estradiol regulation and antioxidative effects were indicators that SWT ameliorated ovarian follicular maldevelopment (Huang et al., 2019). According to Taiwan Herbal Pharmacopeia, ferulic acid is recorded as a quality component in *Angelica sinensis*, and *Ligusticum chuangxiong* with the content of not less than 0.03 and 0.07%, respectively. It is easily soluble in hot water and has also been reported to have antioxidative and anticancer properties, which protect the brain against Alzheimer’s disease and the skin from aging, and prevent the development of cardiovascular disease and diabetes (Lin et al., 2005; Sgarbossa et al., 2015; Gao et al., 2018; Ghosh et al., 2018; Zduńska et al., 2018; Cheng et al., 2019). After intravenous injection of 1 mg of ferulic acid, it was noted that FSH release had increased significantly after 5 min, whereas LH and prolactin release were inhibited at 5 to 10 min. Ferulic acid was noted in LH and prolactin release after injection (Okamoto et al., 1976). This is pertinent to related adverse diseases; however, oxidative factors can interfere with follicular development. Therefore, ferulic acid, with its antioxidant capacity, could be used as an indicator in monitoring different dosage forms of decoctions and concentrated preparations to evaluate whether there is equal efficacy. Nevertheless, many studies have not systematically explained the specific effects of SWT on blood and menstruation. In the case of two different pathological phenomena, how the herbs exert their synergistic or additive effects, with one side having multiple effects is unexplained.

In this study, we cooperated with Taiwan’s good manufacturing practice (GMP) that drug manufacturers use to produce CHEs and used the same source of medicinal materials to prepare a decoction of SWT. To explore the bioequivalence of the two formulations, we used healthy volunteers and performed pharmacokinetic (PK) and pharmacodynamic (PD) studies to observe follicular development. Subjects took both preparations in order to evaluate the bioequivalence of the different formulations. We hope that the results will provide a reference for future clinical use.

## Material and Methods

### Preparation and Quality Control of SWT

#### Preparation of SWT-WD and SWT-CHE

The SWT prescription was based on the unified formula announced by the Committee on Chinese Medicine and Pharmacy of the Department of Health (Taipei, Taiwan) (Ministry of Health and Welfare, 2018). Angelica Radix (*Angelica sinensis* (Oliv.) Diels, *Dang Gui*), Chuanxiong Rhizoma (*Ligusticum chuanxiong* Hort., *Chuan Xiong*), Paeoniae Radix (*Paeonia lactiflora* Pall., *Shao Yao*), and Rehmanniae Radix (*Rehmannia glutinosa* Libosch., *Shu Di Huang*) were purchased from Sun Ten Pharmaceutical (New Taipei City, Taiwan). The medicinal materials were authenticated by the non-profit organization, Brion Research Institute of Taiwan. Voucher specimens (No. AS-20180001 for Angelica Radix, No. LC-20180001 for Chuanxiong Rhizoma, No. PL-20180001 for Paeoniae Radix and No. RG-20180001 for Rehmanniae Radix) was deposited at the College of Pharmacy, Taipei Medical University. The SWT prescription included the four herbs in a 1:1:1:1 ratio. The SWT-WD was prepared using the following method: 600 g of each herb was weighed (total drug weight of 2,400 g), placed in an extractor, and 20 L of filtered water was added and boiled for 30 min until half the volume was left (Tseng et al., 2006). Ten liters of SWT-WD was packaged into 60 packets and the volume of each bag was 170 mL. One packet was used each morning and evening (twice a day). SWT-CHE was purchased from Sun Ten Pharmaceutical (New Taipei City, Taiwan). The batch number of SWT-CHE was 140243. Each 15 g of SWT-CHE contained 8.5 g of extract and 6.5 g of cornstarch conforming to the drug permit license. An 8.5 g extract was derived from the four herbs at a ratio of 1:1:1:1 and each drug was 7.5 g (total drug weight present as 30 g). Based on Sun Ten Pharmaceutical prescription instructions, 30 g of dry herbs was made into 15 g SWT-CHE granules and 18 g of granules was the recommended dose per day. As for the conversion, based on the daily dose of the SWT-CHE we calculated the amount of herbal materials to be 36 g/day. Therefore, the SWT-WD used 170 mL to perform the following experiment, which was the equivalent of 40 g of herbal materials.

#### High-Performance Liquid Chromatography (HPLC) Analysis of Marker Substances in SWT-WD and SWT-CHE

The HPLC apparatus was composed of an SCL-10Avp System Controller, an LC-10ATvp Liquid Chromatograph Pump, an SPD-M10A Diode Array Detector, an SIL-10Avp Auto Injector, a CTO-10A Column Oven, FCV-10Avp Flow-Channel Selection Valves (all from Shimadzu, Tokyo, Japan), and an ERC-3415 Degasser (ERC, Altegolfsheim, Regensburg, Germany). The stationary phase consisted of a Purospher^®^ STAR RP-18e reversed-phase column (5 μm, 4 mm i.d. × 250 mm, Merck, Germany). The mobile phase for chromatographic separation of (A) was 0.05% (v/v) formic acid aqueous solution and (B) acetonitrile using a gradient elution of 12% B at 0–15 min, 17% B at 15.01–30 min, and 100% B at 30.01–60 min. The flow rate was 1.0 mL/min, and the column temperature was maintained at 40°C. We used an ultraviolet wavelength of 320 nm to detect the contents of ferulic acid in SWT-WD and SWT-CHE. An aliquot of 1 g of SWT-CHE powder from the above pharmaceutical company was extracted ultrasonically with 10 mL of water for 1 h at room temperature. The extracted solution was centrifuged at 13,000 rpm for 10 min. The supernatant was collected and diluted 50-fold. Further, SWT-WD was diluted 50-fold with distilled water. The sample solutions (SWT-WD and SWT-CHE) were then filtered through a 0.45 μm filter and 10 μL was directly injected into the HPLC system. Calibration curves were plotted based on the analyte peak areas: y = 11762x − 4380.7, and *R*² = 0.9938.

### PK Study of SWT

#### Subjects

All experimental protocols were reviewed and approved by the Institutional Review Board (IRB) of Taipei Medical University, Taiwan (no.: 100024). This study recruited 23 non-menstrual healthy women (aged 20–35 years). The inclusion criterion was that volunteers could take no nutritional supplements (such as vitamins or iron agents) or drugs (including Chinese and Western medicine) for one week before the experiment.

#### Study Design

The study had an open-label, two group crossover treatment design. Twenty-three volunteers were separated into two groups. In Group A, 14 volunteers were given 18 g of SWT-CHE and 135 mL of SWT-WD. In Group B, 9 volunteers were given 18 g of SWT-CHE and 170 mL of SWT-WD. Volunteers could choose to take SWT-CHE or SWT-WD. A 28-day washout period was observed between doses. For each group, volunteers returned to the clinical facility for subsequent blood drawing for PK sampling.

#### PK Sampling

In each group, blood samples for determination of ferulic acid were collected during confinement at the clinic prior to drug administration and at 0, 10, 30, 60, 120, and 180 min after administration. Five milliliter of blood were collected for each time point from median cubital vein. All blood samples were drawn into blood collection tubes, the interior of which was coated with dipotassium ethylenediaminetetraacetic acid (EDTA).

#### Analytical Methods

Blood samples were cooled in an ice bath and separated by centrifugation at 2000 × *g* for at least 10 min at 4°C. Two aliquots of a minimum of 1 mL plasma were dispensed into polypropylene tubes and maintained at −20 ± 5°C. Plasma samples were used to determine ferulic acid concentrations in human plasma with EDTA as an anticoagulant using an HPLC method. Method validation assays for quantification of ferulic acid in human plasma were carried out according to the currently accepted US Food and Drug Administration (FDA) bioanalytical method validation guidelines. Different concentrations of ferulic acid solutions (10 μL) were spiked into plasma (240 μL) before extraction. First, human plasma (250 μL) was mixed with 125 μL of 0.6 N HCl and vortex-mixed for 10 s. Further, 5,000 μL of ethyl acetate was added for liquid-liquid extraction (Lee et al., 2014). Samples were ultrasonicated for 30 s and centrifuged at 3,000 rpm for 5 min. The supernatant was transferred to a glass test tube and evaporated at 40°C. The residue was dissolved in 250 μL of the mobile phase (0.1% formic acid: acetonitrile, 62:38). The supernatant was filtered through a 0.22 μm mini-filter prior to HPLC analysis. The recovery was assessed by comparing the peak areas of the standard ferulic acid solutions and ferulic acid solutions spiked after extraction into plasma. The validated detection range in human plasma was 1–1,000 ng/mL ferulic acid. The limit of detection (LOD) with a coefficient of variation (CV) of 7.37% at 1 ng/mL. The recovery of ferulic acid from plasma was also estimated at low, medium, and high QC levels (10, 50, and 500 ng/mL). After comparing the peak responses of the spiked samples, the recovery ranged from 92 to 100%.

#### PK Analysis

PK analysis was performed using Phoenix WinNonlin ver. 6.4 (Certara). The maximal measured plasma concentration (C_max_) and the time at which the maximal plasma concentration was observed (T_max_) were taken from the observed plasma concentration-time profile. The area under the concentration-time curve from time zero until the last measurable concentration or the last sampling time, t, whichever occurred first (AUC0-t), was calculated using the trapezoidal method.

### PD Study of SWT in Humans

According to the PK equivalence of the ferulic acid experiment in the first stage, we found that 170 mL of SWT-WD was equivalent to 18 g of SWT-CHE. Therefore, two doses were provided for further PD studies. Twenty-five volunteers were diagnosed at Wan-Fang Hospital, using an ultrasound examination, with an abnormal LH/FSH ratio, abnormal menstrual cycles, and follicular maldevelopment. Together, based on TCM, Dr. Ching-Tzu Lee diagnosed patients who were treated with SWT and who were included in this project. Before taking SWT, blood was taken to check the estradiol (E_2_), FSH, LH, progesterone (P4), testosterone (T) levels, and an ultrasonic examination was performed. Volunteers were randomly selected to take 170 mL of the SWT-WD, which was equivalent to 18 g of the SWT-CHE, according to their own wishes. A parallel design was used. During the experiment, blood was collected each month (if the menstrual period occurred, blood was collected before the 3rd–5th day), and the observation point was at 3 months. At the end of the experiment, follicular development was detected using ultrasound. During the experiment, subjects were not allowed to take other SWT-related products, iron-containing drugs, or other TCM products.

### Statistical Analyses

Results are expressed as mean ± standard deviation. A paired *t*-test was employed to compare differences within the same group (baseline vs. end of treatment), and Student’s *t*-test was used to compare differences between the experimental SWT-WD and SWT-CHE groups. All variables were analyzed *via* a one-way analysis of variance (ANOVA) with a post-hoc least significant difference test. *p* < 0.05 was considered statistically significant. The Statistical Package for the Social Sciences (SPSS) version 17.0 (Chicago, IL, USA) was used for all analyses.

## Results

### Ferulic Acid Contents in SWT-WD and SWT-CHE Groups

**Figure 1:**
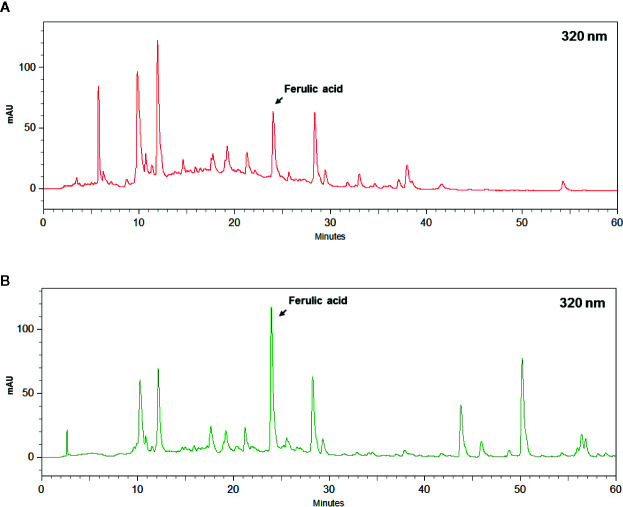
HPLC chromatogram of ferulic acid in **(A)** Si-Wu-Tang (SWT)-water decoction (WD) and **(B)** SWT-concentrated herbal extract (CHE).

According to the above data, the same concentration of ferulic acid in SWT-WD and SWT-CHE were calculated as previously described in the Methods section. Each gram of SWT-CHE contained 440 μg ferulic acid, and each milliliter of SWT-WD contained 58.5 μg of ferulic acid. After calculation, 18 g of SWT-CHE and 135 mL of SWT-WD were determined to have the same amount of ferulic acid (**Figure 1**).

### PK Study of SWT

**Figure 2:**
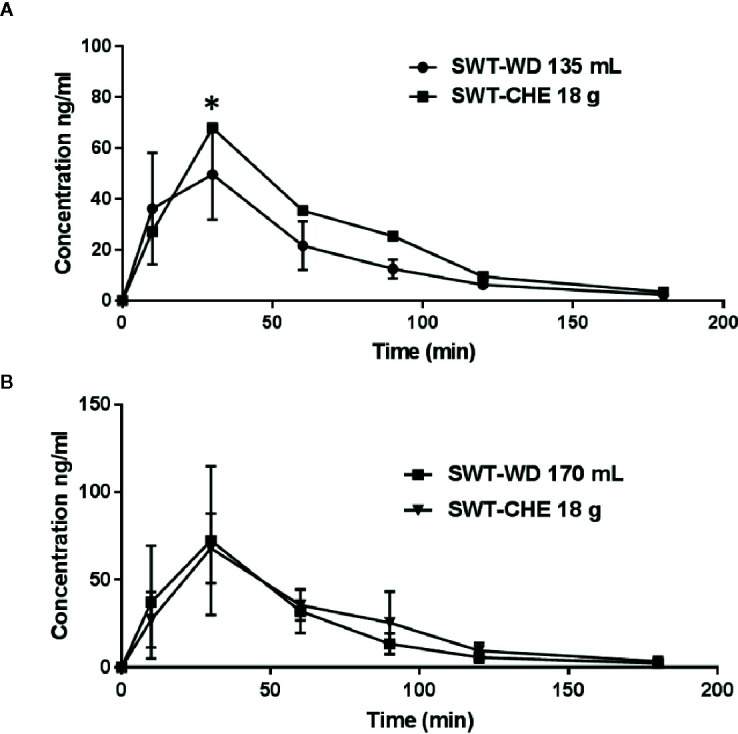
Area under the curve (AUC)-time curve of ferulic acid in the subject’s serum. **(A)** Group with 135 mL of the Si-Wu-Tang (SWT)-water decoction (WD) and 18 g of the SWT-concentrated herbal extract (CHE) and **(B)** the group with 170 mL of SWT-WD and 18 g of SWT-CHE.

The 23 volunteers’ plasma ferulic acid concentration-time courses following a single administration are shown in **Figure 2**. Group A was given 18 g of SWT-CHE and 135 mL of SWT-WD that presented the same amount of ferulic acid. Group B was administered 18 g of SWT-CHE and 170 mL of SWT-WD that presented the clinical dosage. These two groups were used to compare the bioequivalence of ferulic acid. The derived values of the PK parameters are presented in **Table 1**. Results showed that the maximum concentration (C_max_) and the area under the curve (AUC _0-180_ min) of 18 g of SWT-CHE were higher than those with 135 mL of SWT-WD with the same amount of ferulic acid compared to group A. Further, in the clinical dosage group B, C_max_ and the AUC_0-180min_ presented similar data without a significant difference. There was no significant difference between CHE and WD at T_max_ and T_1/2_. Therefore, we chose Group B, 170 mL of SWT-WD and 18 g of SWT-CHE, for using as the clinical dosage for the ovarian follicular maldevelopment PD bioequivalence study.

**Table 1:** Pharmacokinetic parameters of ferulic acid in serum after taking Si-Wu-Tang.

Formulation	Dosage	T_max_ (min)	C_max_ (ng/mL)	AUC_0-180 min_(min × ng/mL)	Cl (mL/min)	T_1/2_ (min)
**A**	135 mL	25.7 ± 8.5^a^	30.9 ± 8.1^a^	3619.7 ± 965.6^a^	2310.4 ± 531.1^a^	131.7 ± 74.5^a^
	18 g	32.9 ± 12.7^a^	42.1 ± 14.2^b^	4404.7 ± 1030.5^b^	1892.7 ± 474.8^b^	88.2 ± 46.7^a^
**B**	170 mL	31.1 ± 12.7^a^	46.6 ± 25.1^b^	4276.2 ± 1140.0^b^	2463.4 ± 642.5^a^	156.9 ± 79.7^a^
	18 g	30.0 ± 0^a^	44.0 ± 11.6^a^	4046.1 ± 752.5^b^	2019.5 ± 409.7^a^	78.4 ± 12.7^b^

T_max_, time at which C_max_ was observed; C_max_, maximal measured plasma concentration; AUC, area under the concentration-time curve; Cl, clearance; T_1/2_, half-life.

### PD Study of SWT

This study was hosted in consultation with Chinese and Western medicinal specialists at Wan-Fang Hospital. Patient inclusion criteria according to clinical conditions were as follows: (1) an LH/FSH ratio of >1, (2) abnormal LH or FSH levels, and (3) follicles <8 mm and their number greater than 12; also a patient mentioning menstrual abnormalities (cycle disorder, or abnormal menstrual blood color or quantity). Twenty-five patients were included in this study; 12 patients underwent SWT-WD and 13 patients underwent SWT-CHE. However, 12 patients withdrew from the process for no given reason, one patient withdrew due to diarrhea (an expected side effect), and one patient changed to another Chinese medicine. Ultimately, six patients completed SWT-WD and five patients completed SWT-CHE.

Before taking SWT, endocrine hormones were determined in all patients with ovarian follicular maldevelopment; FSH and LH levels were found to be significantly higher, and E_2_ levels were found to be significantly lower; this is considered normal levels for the menopausal period. Due to the low level of estrogen, the level of emergence of bottom cells was also low.

**Figure 3:**
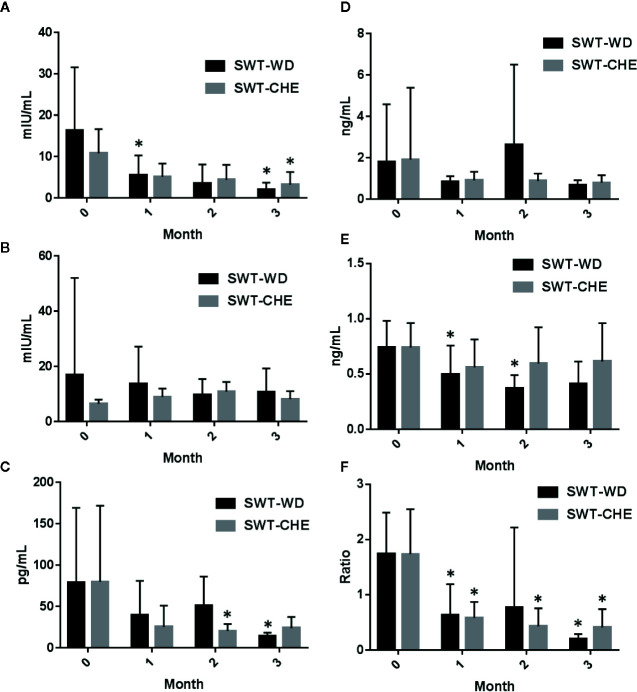
**(A)** Luteinizing hormone (LH), **(B)** follicle-stimulating hormone (FSH), **(C)** estradiol, **(D)** progesterone, **(E)** testosterone, and **(F)** the LH/FSH ratio in the serum of subjects in the Si-Wu-Tang (SWT)-water decoction (WD) and SWT-concentrated herbal extract (CHE) treatment groups.

After taking SWT-WD or SWT-CHE, the LH of patients was significantly lower in the first month. As the duration of taking SWT increased, the effect was more significant (**Figure 3A**) and there was no significant difference between SWT-WD and SWT-CHE. In the estradiol analysis, SWT-WD reduced estradiol levels by the third month and SWT-CHE reduced estradiol levels by the second month (**Figure 3C**). However, the FSH and progesterone levels of patients exhibited no significant difference before and after taking SWT-WD or SWT-CHE (**Figures 3B, D** **)**. Taking SWT-WD reduced testosterone levels in the first and second months. The LH/FSH ratio of all six patients was <1 and the value returned to normal after the study (**Figure 3F**). In the SWT-CHE group, the LH/FSH ratio of four patients was <1. However, the LH/FSH ratio was still abnormal, as observed in one patient and did not return to a normal range after the study.

## Discussion

Ferulic acid, a quality component of *A. sinensis*, and *L. chuangxiong*, has also been used as a quality component to evaluate the amount in SWT-WD and SWT-CHE. Based on the analysis of data from group A, 135 mL of SWT-WD and 18 g of SWT-CHE represented the same amount of ferulic acid, and according to Sun Ten Pharmaceutical prescription instructions, 170 mL of SWT-WD and 18 g of SWT-CHE represented the clinical dosage. However, when 18 g of SWT-CHE was taken, the AUC of ferulic acid was higher than that with 135 mL of SWT-WD, but was similar to that with 170 mL of SWT-WD. In the experiment with equal amounts of ferulic acid, SWT-CHE presented a higher C_max_ and AUC than SWT-WD. This showed the superiority of the concentrated extract with a high blood concentration. According to **Figure 2** and **Table 1**, FA was shown quickly absorbed after oral administration and reaches the peak plasma concentration (C_max_) around 30 min (T_max_). Ferulic acid was metabolism in the liver through CYP 1A1 and 2B7 of the UDP-glucuronosyl-transferase (UGT) (Li, X., Shang, L., Wu, Y., Abbas, S., Li, D., Netter, P., Ouzzine, M., Wang, H., Magdalou, J., 2011. Identification of the human UDP-glucuronosyltransferase isoforms involved in the glucuronidation). As clinical studies, glucuronide (3–20%) and sulfo-glucuronide (60–90%) are the most abundant metabolites of FA in plasma, whereas only a low percentage of unmodified FA (9–20%) has been found. FA and its metabolites are excreted mainly from the kidney. (Zhao et al., 2003; Zhao et al., 2004). Our results showed that both SWT-CHE and SWT-WD presented similar characteristic.

Chinese medicine treatment for infertility is divided into five major types of diseases: kidney yang-deficiency, kidney yin-deficiency, liver qi stagnation, blood stasis, and phlegm, and dampness types (Hsu et al., 2006). According to the results, both formulations of SWT could ameliorate follicular maldevelopment and irregular menstrual cycles. It was speculated that patients with abnormal LH and FSH levels may have liver qi stagnation. When adjusting the daily dose of SWT for 3 months, either taking SWT-WD or SWT-CHE, both formulations produced sufficient blood concentrations of ferulic acid, which could improve ovarian follicular maldevelopment. It has been reported that oxidation could be a reason for causing failure of ovary function (Wang et al., 2018). Therefore, both agents presented equivalent effects. Taken together, ferulic acid is a good indicator to analyze the bioequivalence in both PK and PD studies. Despite the preclinical evidence supporting the potentially important role of ferulic acid in free radical-induced diseases. Whereas only a few clinical studies were performed keeping in mind a pharmacodynamic target or a specific disease. Our experiment showed that both SWT-CHE and SWT-WD with same amount ferulic acid could improve ovarian follicular maldevelopment.

**Figure 4:**
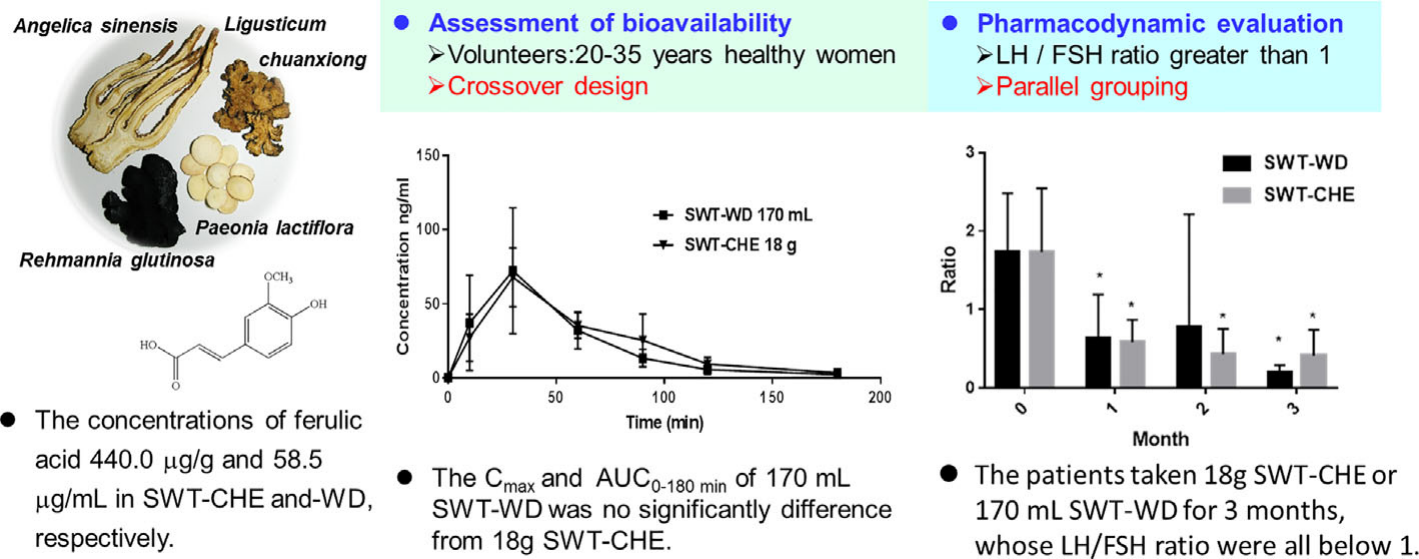
Summary of the pharmacokinetic/pharmacodynamic studies of Si-Wu-Tang (SWD).

However, from current clinical experience, doctors usually advise patients to use a decoction once in the morning and evening. According to our experiment, taking SWT-WD once a day, has already presented the same blood concentration as SWT-CHE and showed good amelioration of follicular maldevelopment. Therefore, the common daily dose of the decoction was twice that of the concentrated extract. Patients that have previously thought that the decoction was better than the concentrated extract may just have taken more drug than the concentrated extract. Our results showed that WD and CHE, both prepared from 40 g of SWT, displayed bioequivalence upon PK/PD analysis. Based on the results of this study, we recommend that the clinical dosage used in these two formulations should be considered as an equivalent amount between the PK and PD experiments (**Figure 4**). In conclusion, Si-Wu-Tang could improve ovarian follicular maldevelopment. But there are still many ingredients and related to the efficacy need to be extensively explored and discussed in future study.

## Data Availability Statement

The raw data supporting the conclusions of this article will be made available by the authors, without undue reservation, to any qualified researcher.

## Ethics Statement

The studies involving human participants were reviewed and approved by Institutional Review Board (IRB) of Taipei Medical University, Taiwan (no.: 100024). The patients/participants provided their written informed consent to participate in this study.

## Author Contributions

C-JL and C-CW conceived and designed the experiments. C-JL, AK, Y-ZT, and C-TL performed the experiments. C-JL and M-YX analyzed the data. C-JL wrote the manuscript. C-CW revised the manuscript.

## Funding

This work was financially supported by the by Taipei Medical University (Grant Number: TMU103-AE1-B38).

## Conflict of Interest

The authors declare that the research was conducted in the absence of any commercial or financial relationships that could be construed as a potential conflict of interest.
